# Phenotypic characterization of Familial Mediterranean Fever patients harboring variants of uncertain significance

**DOI:** 10.3906/sag-2011-273

**Published:** 2021-08-30

**Authors:** Alper SARI, Erdal BODAKÇİ, Berkan ARMAĞAN, Hasan SATIŞ, Nuh ATAŞ, Nazife Sule YAŞAR BİLGE, Reyhan BİLİCİ SALMAN, Gözde Kübra YARDIMCI, Hakan BABAOĞLU, Levent KILIÇ, Mehmet Akif ÖZTÜRK, Şeminur HAZNEDAROĞLU, Berna GÖKER, Umut KALYONCU, Timuçin KAŞİFOĞLU, Abdurrahman TUFAN

**Affiliations:** 1 Department of Rheumatology, Faculty of Medicine, Hacettepe University, Ankara Turkey; 2 Department of Rheumatology, Faculty of Medicine, Osmangazi University, Eskisehir Turkey; 3 Department of Rheumatology, Faculty of Medicine, Gazi University, Ankara Turkey

**Keywords:** Familial Mediterranean Fever, MEFV, variants of uncertain significance, genetics, phenotype

## Abstract

**Background/aim:**

Familial Mediterranean Fever (FMF) is the prototype of hereditary autoinflammatory disorders and caused by mutations on the MEFV gene located on the short arm of chromosome 16. Although some MEFV variants are clearly associated with disease phenotype, there are numerous variants with unknown clinical association which are termed as variants of uncertain significance (VUS). Here, we present clinical correlations of VUS in a large cohort of adult FMF patients from three tertiary centers located in Central Anatolia.

**Materials and methods:**

All patients were recruited from FMF in Central Anatolia (FiCA) cohort. Demographic (sex, age at disease onset) and clinical features (disease characteristics, attack frequency, mean colchicine dose, colchicine nonresponsiveness, amyloidosis, and persistent inflammation) of patients with VUS were compared with those harboring pathogenic variants. Disease severity and damage were also evaluated using international severity score for FMF (ISSF) and autoinflammatory disease damage index (ADDI), respectively.

**Results:**

Among 971 participants included, MEFV gene analysis results were available for 814 patients. Twenty-six (3.2%) patients had single heterozygous VUS and 54 (6.6%) had pathogenic/VUS complex heterozygous variants. Patients with single heterozygous VUS had similar demographic/clinical features, ISSF and ADDI scores compared to those with single heterozygous pathogenic variant (p > 0.05 for all). No difference was observed in the demographic and clinical features of patients with single heterozygous pathogenic mutation and pathogenic/VUS complex heterozygous variant (p > 0.05 for all). ISSF and ADDI scores were lower in pathogenic/VUS complex heterozygous patients than those harboring single pathogenic mutation (p = 0.006 and 0.004, respectively).

**Conclusion:**

Our findings suggest that patients with single heterozygous VUS has mild FMF phenotype similar to those with single pathogenic mutation. Pathogenic/VUS complex heterozygosity does not lead to a more severe clinical phenotype than having a single pathogenic variant.

## 1. Introduction

Familial Mediterranean fever (FMF) is the most common monogenic autoinflammatory disease (AID) worldwide [1]. Although it has the highest prevalence among people originated from Eastern Mediterranean, can also be recognized in subjects from different ethnicities [2–4]. FMF is caused by mutations in the MEFV gene which is located on chromosome 16 [5]. MEFV gene is composed of 10 exons and encodes a 781–amino acid protein called pyrin [6]. Pyrin plays a key role in innate immunity and when mutated, leads to an exaggerated inflammation through abundant release of interleukin-1β [7]. FMF follows an autosomal recessive pattern of inheritance, however, classic phenotypic characteristics may exist in almost 30% of patients who are single heterozygous [8,9]. To date, more than 300 variants have been identified within the MEFV gene region^1^
http://fmf.igh.cnrs.fr/infevers/.. These variants are classified as benign, likely benign, likely pathogenic and pathogenic; according to their potential association with disease phenotype with current evidence [10]. However, there are numerous variants with unknown clinical association which are termed as variants of uncertain significance (VUS) [10,11]. These variants could be found either homozygous or single or complex heterozygous. Impact of these variants on final clinical phenotype and disease complications needs to be elucidated for proper management of patients. In this study, we aimed to investigate the clinical significance of VUS in a large multicenter cohort of Turkish FMF patients mainly originated from Central Anatolia. 

## 2. Materials and methods

FMF in Central Anatolia (FiCA) is a cross-sectional, multicenter web-based cohort consisting of adult (≥ 18 years old) FMF patients admitted to outpatient rheumatology departments of three tertiary referral centers located in central Turkey between January and December 2018. All recorded patients fulfilled the Tel-Hashomer classification criteria for FMF and had at least 6 months of follow-up [12]. Data obtained from patient interviews included demographics, disease and treatment characteristics, comorbidities and disease related complications. Laboratory, pathologic and genotype data were collected from hospital records. The diagnosis of amyloidosis was confirmed with tissue biopsy in all suspected cases.

### 2.1. Definitions and patient assessments

Persistent inflammation was defined as an increased serum C-reactive protein (CRP) levels during the attack-free period (at least 2 weeks apart from attack) and in more than 75% of follow-up visits was [13]. We defined colchicine nonresponsiveness as having more than one attack per month for 3 months duration despite the use of maximal tolerated dose of colchicine [14].

Disease severity and FMF related damage were assessed using the international severity score for FMF (ISSF) [15] and autoinflammatory disease damage index (ADDI), respectively [16]. Briefly, ISSF consists of nine clinical and laboratory variables: chronic sequela, organ dysfunction, organ failure, attack frequency, increased acute-phase reactants, involvement of more than two sites during an individual acute attack, more than two different types of attack during the course of the disease, duration of attacks, and exertional leg pain. The maximum score is 10 and the degree of severity was determined as mild (≤2), intermediate (3–5) or severe disease (≥6) [15]. In ADDI, damage is defined as “persistent or irreversible change in structure or function, that is present for more than 6 months”. ADDI consists 18 items, and these items are categorized by organ systems as follows: reproductive, renal/amyloidosis, developmental, serosal, neurological, ears, ocular and musculoskeletal. The renal/amyloidosis and neurological damage categories were assigned to have the highest number of points while serosal damage got the lowest. This index provides a universal instrument to measure damage by chronic inflammation in FMF [16].

### 2.2. Genetic analysis

The MEFV gene variants were genotyped by pyrosequencing and direct Sanger sequencing techniques. The 22 common variants profiles; E148Q, R202Q, P369S, H478Y, F479L, S675N, G678E, M680L, M680I(G>A & G>C), T681I, I692DEL, M694V, M694I, M694L, K695N, K695R, I720M, V722M, V726A, A744S, R761H were genotyped by pyrosequencing. Some patients who had clinical features without mutated pyrosequencing profiles were genotyped for exon 10 by direct sequencing analysis. VUS variants were E148Q, P369S, H478Y, G678E, T681I, I720M, V722M, A744S. Pathogenic variants were F479L, M680L, M680I, I692DEL, M694V, M694I, M694L, K695N, K695R, V726A, and R761H^2^ https://infevers.umai-montpellier.fr/. Likely pathogenic variants were classified as pathogenic variant in this study. Patients were grouped based on different combinations of MEFV variants in two alleles;

1) Mutation negative (-/-)

2) Single pathogenic (M694V/-, M680I/-, etc.)

3) Single VUS (E148Q/-, A744S/-, etc.)

4) Biallelic (double) pathogenic, homozygous or complex heterozygous (M694V/M694V, M680I/V726A, etc.)

5) Pathogenic and VUS complex heterozygous (M694V/E148Q, M680I/A744S etc.).

### 2.3. Statistical analysis

Statistical analysis was performed using SPSS Statistics for Windows v: 21.0 (Chicago, IL, USA). Categorical variables were expressed as number and percentage. Continuous variables were expressed as mean (standard deviation, SD) for normally distributed and median (interquartile range, IQR) for skewed data. Chi-square and Fisher’s exact test were used to compare categorical data. For normally distributed continuous variables, Student’s t test was used to compare the means between two groups and one-way ANOVA was used to compare the means among 3 groups. Mann–Whitney U and Kruskal–Wallis tests were used for comparison of non-normally distributed continuous data between two and three groups, respectively. We used Bonferronni correction for posthoc analysis after ANOVA while intergroup comparisons were performed with Mann–Whitney U test after Kruskall–Wallis test. In either condition significance level was set at <0.0167 for posthoc analysis.

## 3. Results

Among 971 (61.5% female) FMF patients enrolled in whole cohort, MEFV gene analysis results could be obtained for 814 subjects (60.9% female). Median age at study enrollment, symptom onset and FMF diagnosis were 34 (25–43), 10 (6–18) and 24 (15–33) years, respectively. Median disease duration from the onset of symptoms was 20 (12–29) years. Peritonitis was the most common clinical feature and present in 90.4% of patients followed by fever (82.1%), pleuritis (49.0%), arthritis (44.2%), erysipelas-like erythema or purpuric rash (27.3%), and myalgia (24.1%). One hundred and twenty-eight (15.7%) patients had persistently elevated acute phase response and 50 (6.1%) had amyloidosis. Mean colchicine dose was 1.3 (0.5) mg/day and 72 (8.8%) patients were classified as colchicine nonresponsive. Median ISSF score was 3 (2–4) and disease severity categories according to ISSF among patients were as follows: mild disease in 45.2%, moderate disease in 47.3% and severe disease in 7.5% of patients. Using the ADDI index, more than half of patients (n = 482, 59.2%) had disease related damage. 

At least one MEFV variant was present in 769 (94.5%) patients. M694V was the most frequent variant with being present in 618 (75.9%) patients. 259 (31.8%) and 423 (51.9%) patients had single and biallelic pathogenic mutations respectively, without harboring any VUS. 26 (3.2%) patients had single VUS (E148Q in 21, A744S in 4 and P369S in 1 patient). VUS and pathogenic complex heterozygosity was present in 54 (6.6%) patients. Among these patients, 47 had E148Q and 7 had A744S variant. 3 patients had biallelic VUS; 2 of them had P369S/E148Q complex heterozygous variant and the other patient had homozygous E148Q. Allelic frequency of MEFV gene variants are summarized in Table 1. 

**Table 1:** Allelic frequencies of common MEFV gene variants excluding mutation negative subjects (n = 769).

Pathogenic variants	N (%)	VUS variants	N (%)
M694V	844 (54.9)	E148Q	77 (5.0)
M680I	185 (12.0)	P369S	4 (0.3)
V726A	107 (6.9)	A744S	11 (0.7)
R761H	21 (1.4)		
F479L	7 (0.4)		
K695R	2 (0.1)		

No difference in demographics, clinical features, disease severity, and FMF related damage was observed among patients with single VUS, single pathogenic mutation, and no mutation (Table 2). Among 285 subjects with single MEFV mutation, patients with M694V variant (n = 207) had more frequent arthritis, persistent inflammation, and amyloidosis than those without (data not shown). There was no significant difference for any characteristics between patients with single VUS and single non-M694V pathogenic variants (Table 3).

**Table 2:** Characteristics of patients with single pathogenic mutation, single VUS and no mutation.

	Single pathogenic(n = 259)	Single VUS(n= 26)	Mutation negative(n = 45)	p	p1	p2	p3
Female	153 (59.1)	18 (69.2)	30 (66.7)	0.41	0.31	0.82	0.33
Age at symptom onset, years	15 (9.0–23.0)	17.5 (10.0–26.2)	15 (8.0–24.5)	0.70	0.41	0.49	0.86
Number of attacks during the last year	2 (0–5)	1.5 (0–4)	2 (0–4)	0.50	0.49	0.90	0.30
Fever	201 (77.6)	23 (88.5)	33 (73.3)	0.32	0.19	0.13	0.53
Peritonitis	227 (87.6)	23 (88.5)	39 (86.7)	0.97	1.000	1.000	0.85
Pleuritis	96 (37.1)	8 (33.3)	15 (33.3)	0.84	0.71	1.000	0.63
Arthritis	96 (37.1)	5 (20.0)	11 (24.4)	0.07	0.08	0.67	0.09
Myalgia	42 (16.2)	4 (16.7)	6 (13.3)	0.88	1.000	0.73	0.62
Persistent inflammation	24 (9.6)	-	1	0.07	0.14	1.000	0.14
Colchicine dose, mg/d, mean	1.2 (0.5)	1.1 (0.5)	1.1 (0.6)	0.69	0.54	0.86	0.59
Colchicine non-responsiveness	15 (5.8)	1	2	0.87	1.000	1.000	1.000
Amyloidosis	14 (5.4)	0	2	0.46	0.62	0.52	1.000
ADDI score	1 (0–1)	1 (0-1)	1 (0–1)	0.40	0.98	0.34	0.18
Any damage in ADDI	156 (60.2)	16 (61.5)	23 (51.1)	0.49	0.89	0.39	0.25
ISSF	2 (2-3)	2 (1-3)	2 (1-3)	0.12	0.28	0.75	0.06
ISSF categoryMild diseaseModerate/severe disease	139 (53.7)120 (46.3)	15 (57.7)11 (42.3)	30 (66.7)15 (33.3)	0.26	0.69	0.45	0.10

**Table 3:** Characteristics of patients harboring single MEFV variant with respect to their potential penetrance.

	Single M694V(n = 207)	Single non-M694V pathogenic (n = 52)	Single VUS (n = 26)	p
Age	36.2 (13.1)	36.0 (11.3)	36.1 (10.8)	0.91
Female	121 (58.4)	32 (61.5)	18 (69.2)	0.63
Age at symptom onset, years	15 (10–25)	14 (7–23)	18 (10–26)	0.94
Number of attacks within the last year	2 (0–5)	3 (0–6)	2 (0–4)	0.46
Fever	159 (76.8)	42 (80.8)	23 (88)	0.40
Peritonitis	184 (89)	43 (83)	23 (88)	0.50
Pleuritis	77 (37)	19 (37)	8 (33)	0.97
Arthritis	84 (41)	12 (23)	5 (20)	0.016
Skin rash	41 (20)	1 (2)	2 (8)	0.001
Myalgia	33 (16)	9 (17)	4 (17)	0.96
Persistent inflammation	24 (12)	0	0	0.007
Colchicine dose, mg/d, mean	1.2 (0.5)	1.2 (0.4)	1.1 (0.5)	0.71
Colchicine nonresponsiveness	14 (6.8)	1 (1.9)	1 (4)	0.37
Amyloidosis	14 (6.8)	0	0	0.01
ADDI score	1 (0–1)	1 (0–1)	1 (0–1)	0.45
ISSF score	2 (2–3)	3 (2–3)	2 (1–3)	0.70
ISSF severe disease	13 (6.3)	1 (1.9)	0	0.39

Compared to patients with biallelic pathogenic mutations, complex heterozygous patients harboring pathogenic mutation(s) and VUS had older age at disease onset, lower number of attacks during last year, lower mean colchicine dose and lower median ISSF and ADDI scores (Table 4). These patients also less frequently had pleuritis, arthritis, myalgia, persistent inflammation, colchicine nonresponsiveness, moderate/severe disease course, and any disease-related damage than patients with biallelic pathogenic mutations. Patients with single pathogenic mutation had higher ISSF and ADDI scores and more frequently had moderate/severe disease and disease related damage compared to patients harboring pathogenic and VUS complex heterozygous variant.

**Table 4:** Characteristics of patients with biallelic pathogenic mutation, pathogenic/VUS complex heterozygous mutation and single pathogenic mutation.

	Biallelic pathogenic (homozygous or complex heterozygous) (n= 423)	Pathogenic and VUS complex heterozygous(n = 54)	Single pathogenic(n = 259)	p	p1	p2
Female	259 (61.2)	31 (57.4)	153 (59.1)	0.77	0.58	0.82
Age at symptom onset, years	8 (5–13)	16 (11–25)	15 (9–23)	<0.001	<0.001	0.10
Number of attacks during the last year	3 (1–8)	2 (0–4)	2 (0–5)	0.001	0.040	0.90
Fever	364 (86.1)	42 (77.8)	201 (77.6)	0.008	0.08	0.97
Peritonitis	393 (92.9)	47 (87.0)	227 (87.6)	0.026	0.10	0.90
Pleuritis	258 (61.0)	18 (33.3)	96 (37.1)	<0.001	<0.001	0.66
Arthritis	227 (53.7)	17 (31.5)	96 (37.1)	<0.001	0.002	0.42
Myalgia	135 (31.9)	7 (13.0)	42 (16.2)	<0.001	0.006	0.61
Persistent inflammation	98 (23.2)	5 (9.3)	24 (9.6)	<0.001	0.019	1.000
Colchicine dose, mg/d, mean	1.4 (0.5)	1.0 (0)	1.2 (0.5)	<0.001	<0.001	0.06
Colchicine nonresponsiveness	53 (12.5)	1 (1.9)	15 (5.8)	0.002	0.020	0.32
Amyloidosis	32 (7.6)	2 (3.7)	14 (5.4)	0.36	0.40	1.000
ADDI score	1 (0–2)	0 (0–1)	1 (0-1)	0.001	0.001	0.004
Any damage in ADDI	261 (61.8)	23 (42.6)	156 (60.2)	0.025	0.007	0.017
ISSF	3 (2–4)	2 (1–3)	2 (2–3)	<0.001	<0.001	0.006
ISSF categoryMild disease Moderate/severe disease	141 (33.3)282 (66.7)	37 (68.5)17 (31.5)	139 (53.7)120 (46.3)	<0.001	<0.001	0.045

## 4. Discussion

In the present study, we analyzed the association of MEFV variants with uncertain significance with clinical phenotype in a multi-center large cohort consisted of Turkish patients with FMF. Results of our study disclosed that patients with single heterozygous VUS variants have a similar disease course as those with the single pathogenic variants. Moreover, we found that, complex heterozygous patients with pathogenic variant and VUS have an attenuated disease phenotype characterized by milder disease course and reduced risk of disease complications.

Genetic tests have been implemented in the diagnosis of autoinflammatory diseases for a long time [17]. However, despite a few pathogenic variants being intensively studied, literature data about the genotype-phenotype correlation of most MEFV variants remain inconclusive. Recently, a consensus based pathogenicity classification of MEFV variants was proposed [18]. Although pathogenicity of some variants agreed by consensus of experts, a large number of MEFV variants were classified as VUS or “unsolved pathogenicity”. This highlighted the need for better characterization of the impact of these variants on the clinical phenotype.

Vast majority of patients with VUS in our cohort had E148Q variant which is a prevalent mutation in MEFV allele [18,19]. Whether E148Q is polymorphism or disease-causing mutation has been highly debated. Ben-Chetrit et al. observed similar frequency of E148Q variant both in patients and healthy controls and concluded E148Q as a benign polymorphism [20]. The fact that the functional response evaluated by ex-vivo colchicine assay is similar between patients with E148Q and healthy controls expressing wild-type pyrin supports this view [21]. However, some other studies demonstrated that patients with homozygous E148Q variant may develop FMF-like illness [22]. On the other hand, data about clinical phenotype of patients with heterozygous E148Q variant is limited and controversial. Our results showed no difference in clinical features, disease severity and damage between patients with single heterozygous pathogenic and single heterozygous VUS variants. Most of these similarities, except arthritis, persisted when patients with heterozygous VUS were compared with those with single heterozygous M694V. These results should be carefully interpreted as there were no patient with amyloidosis in the VUS group while about 5% of patients with heterozygous pathogenic variant had amyloidosis. One study on children with periodic fever and carrying MEFV mutations reported that patients with heterozygous E148Q or V726A variant less frequently experienced severe abdominal and chest attacks compared to those with heterozygous exon 10 mutations (M694V, M694I, M680I) [23]. More stringent classification criteria used in our study might have led to selection of more severe patients with heterozygous VUS variant. In line with our findings, Kilic et al. reported similar disease severity between patients harboring heterozygous exon 2 and exon 10 mutations in a cohort of FMF patients classified according to Tel-Hashomer criteria [24].

Interestingly, patients with pathogenic and VUS complex heterozygous variant had similar clinical features with those who had single pathogenic mutation. Moreover, severity and damage scores were lower in pathogenic and VUS complex heterozygosity. These findings suggest that VUS may not have an additive effect on clinical phenotype when present together with a pathogenic mutation. Very few studies in the literature provided information about this issue and had conflicting results mostly focusing on E148Q which is a relatively frequent variant [25,26]. Occasional reports suggested that E148Q may have an aggravating effect when present as a part of complex allele with V726A or M694I [27,28]. On the other hand, a recent study reported that subjects with exon 10 and non-exon 10 complex heterozygous variant had similar clinical features and amyloidosis frequency compared to patients with single heterozygous mutation [29]. However, results of that study were not suitable to compare with ours as single heterozygous group included both VUS and pathogenic exon 10 mutations. Due to controversial results in literature, we think in vitro functional studies are needed to elucidate how VUS genotype contribute to clinical phenotype when harbored in combination with clearly pathogenic mutations [21].

This study was conducted in one of the largest adult FMF cohort with considerable amount of patients with VUS. Retrospective design and lack of in vitro functional evaluation are the main limitations to be addressed. Due to small number of patients in specific VUS variants, we could not characterize phenotypic effect of each particular VUS genotype. Small number of patients with VUS other than E148Q (P396S and A744S) did not allow us to draw any specific conclusions on these variants and also limited the generalizability of our results.

In conclusion, harboring a single VUS results in a mild FMF phenotype similar to those observed in patients with single heterozygous pathogenic variant. Pathogenic/VUS complex heterozygosity does not lead to a more severe clinical phenotype than having single heterozygous pathogenic variant.

## Informed consent

Non-Interventional Clinical Research Ethics Committee of Gazi University approved this study (2017-622).
